# Evolution of Thoracic Disc Herniation Surgery: Future Perspectives from a Systematic Review and Meta-Analysis

**DOI:** 10.3390/brainsci14111062

**Published:** 2024-10-25

**Authors:** Giuseppe Corazzelli, Sergio Corvino, Giulio Di Noto, Chiara Di Domenico, Federico Russo, Giuseppe Mariniello, Andrea Elefante, Antonio Bocchetti, Sergio Paolini, Vincenzo Esposito, Gualtiero Innocenzi, Raffaele de Falco, Oreste de Divitiis

**Affiliations:** 1 Department of Neurosciences and Reproductive and Odontostomatological Sciences, Neurosurgical Clinic, University of Naples “Federico II”, 80131 Naples, Italy; sercorvino@gmail.com (S.C.); didomenico.chiara96@gmail.com (C.D.D.); dr.federicorusso@gmail.com (F.R.); giuseppe.mariniello@unina.it (G.M.); 2 Neurosurgery Department, Santa Maria delle Grazie Hospital, ASL Napoli 2 Nord, 80078 Naples, Italy; antonio.bocchetti74@gmail.com (A.B.); raffaele.defalco@aslnapoli2nord.it (R.d.F.); 3 Division of Neurosurgery, Università degli Studi di Messina-Policlinico “G. Martino”, 98124 Messina, Italy; giuliodinoto@libero.it; 4 Department of Advanced Biomedical Sciences, University of Naples “Federico II”, 80138 Naples, Italy; aelefant@unina.it; 5 Department of Neurosurgery, IRCCS Neuromed, 86077 Pozzilli, Italy; sergio.paolini@uniroma1.it (S.P.); vincenzo.esposito@uniroma1.it (V.E.); innocenzigualtiero@tiscali.it (G.I.)

**Keywords:** thoracic disc herniation, posterolateral approach, anterior approach, minimally invasive surgery, spinal surgery, meta-analysis, surgical complications

## Abstract

Background: The neurosurgical treatment of thoracic disc herniation (TDH) has undergone dramatic changes over the years in terms of surgical approaches and intraoperative technological tools. There is still no unanimous consent on the criteria for approach selection, and the choice varies among Institutions. The aim of this study is to compare anterior and posterolateral approaches for TDH in terms of functional and surgical outcomes. Methods: A systematic literature review and meta-analysis according to PRISMA guidelines from EMBASE, PubMed, Cochrane Library, Web of Science, and Google Scholar online databases up to May 2024 incorporated studies that reported outcomes of thoracic disc herniation surgeries. Analyzed factors included major peri- and postoperative complications, intraoperative blood loss, hospital stay, neurological improvement, and complete hernia resection. Random-effect models were used to calculate pooled odds ratios and mean differences. Results: The posterolateral approach was associated with significantly lower rates of major medical (OR 0.14, 95% CI: 0.07 to 0.27) and surgical complications (OR 0.61, 95% CI: 0.38 to 0.99) compared to the anterior approach. Additionally, posterolateral approaches reduced intraoperative blood loss and shorter hospital stays. Posterolateral techniques were linked to higher odds of neurological improvement (OR 0.65, 95% CI: 0.43 to 0.99) and higher rates of complete hernia resection (OR 0.38, 95% CI: 0.21 to 0.71). Conclusions: Posterolateral approaches offer advantages in terms of safety, recovery, neurological improvement, and complete hernia resection. More extensive prospective studies are needed to confirm these findings and refine surgical strategies. Emerging technologies, such as the exoscope and 45° endoscopy, may further enhance surgical outcomes.

## 1. Introduction

Among numerous and different neoplastic [1,2,3,4], infectious–inflammatory [5,6], vascular [7], degenerative [8,9], and traumatic diseases that can affect the spine, disc herniation is one of the most frequently encountered in daily clinical practice. In this setting, the thoracic segment is involved in less than 1% of disc herniations of all spinal segments, with an overall incidence of 1 per 1,000,000 patient-years [10]. Most thoracic disc herniations (TDHs) are central or central–lateral and, less frequently, are truly lateral; calcifications are reported in 30 to 70% of cases [10]. These data are very important from the surgical point of view as the selection of approach mainly depends on two characteristics: the consistency and the location of the disc herniation in the axial plane within the spinal canal. Commonly, anterior approaches are recommendable for disc herniations on the midline and/or hard in consistency. Conversely, paramedian or lateral disc herniations, and/or soft in consistency, are more suitable for posterior or posterolateral approaches, including lateral extracavitary, transfacet, transpedicular, and transforaminal [8]. Nevertheless, there is no unanimous consent on the criteria for the selection of an approach with a prevalence of the anterior/anterolateral route based on historical data.

The neurosurgical treatment of TDHs has undergone dramatic changes over the years in terms of surgical corridors and techniques, as well as technologies adopted.

Initially, starting from the first reported surgical procedure by Mixter and Barr in 1934 [11], the discectomy was performed via standard dorsal laminectomy; nevertheless, this approach yielded high morbidity rates of 18 to 75% and mortality rates up to 50% [12,13,14]. As a result, to overcome this limit, surgeons began to explore alternative ventral routes to avoid the obstacle of the thecal sac and achieve promising results.

In addition, we witnessed the progressive and continuous refinements in the technologies and their application to spine surgery, increasingly aiming at mini-invasiveness, starting from the adoption of the operative microscope in the 1960s through to the endoscope in the 1990s until the last advance represented by the high-resolution 3D exoscope [15,16,17,18,19,20,21].

Therefore, currently, anterior, anterolateral, posterolateral, and posterior, open microsurgical, endoscopic-assisted, purely endoscopic, and exoscopic approaches are in the armamentarium of the neurosurgeon, each with related advantages and limits [22].

The aim of this study was to compare the safety and efficacy of anterior and posterolateral approaches for TDH. Anterior and posterolateral approaches were selected for this review as they are the most commonly used and viable for thoracic disc herniation (TDH) surgery, with extensive documentation in the literature. The anterior approach is suited for central, calcified herniations, while the posterolateral approach is preferred for lateral herniations. Other approaches were excluded due to their lower frequency and increased technical complexity, which would have introduced heterogeneity. The primary outcome was the incidence of major complications, including both medical and surgical events. Secondary outcomes included intraoperative blood loss, postoperative hospital stay, surgical time, neurological improvement, and uncompleted hernia resection. While various scales exist for assessing spinal injuries, the Frankel scale was chosen for this review because it has been historically utilized in our institution, and many of the included studies relied on this scale, ensuring consistency in data reporting [23]. Although the ASIA scale is widely recognized internationally, our aim was to align with the methodologies used in the studies we reviewed [23]. This review compared the anterior and posterolateral approaches for TDH to clarify their general outcomes. While approach selection is case-dependent, this comparison provided guidance when either option is feasible. Odds ratios (ORs) were calculated for neurological improvement and uncompleted hernia resection. This manuscript presents a comprehensive systematic review and meta-analysis to evaluate these outcomes.

## 2. Materials and Methods

### 2.1. Literature Search Strategy

The systematic review was conducted in accordance with the Preferred Reporting Items for Systematic Reviews and Meta-Analyses (PRISMA) guidelines. The literature search was performed across PubMed, EMBASE, Cochrane Library, Web of Science, and Google Scholar for studies published from 1990 to May 2024. The 1990 cut-off was chosen to ensure that the studies included reflected the modern era of TDH surgery [8], following advancements in imaging technologies and surgical techniques that became widely adopted around this time [24]. The search included studies reporting surgical outcomes of TDH surgeries. The keywords used were “Thoracic disc herniation”, “Adult spinal surgery”, “Posterolateral approach”, “Anterior approach”, “Thoracotomy”, and “Thoracoscopy”, with Medical Subject Headings (MeSH) terms and Boolean operators (AND/OR) applied. Our search strategy used broader MESH terms such as ‘Thoracic Disc Herniation’, ‘Posterolateral Approach’, and ‘Anterior Approach’ to capture a wide range of studies. While we considered more specific terms (e.g., ‘costotransversectomy’, ‘trans-pedicular approach’), we excluded them due to their limited representation in the majority of available studies. This decision helped us ensure that the review remained comprehensive, avoiding the exclusion of studies that used less specific terminology. The search was limited to studies published in English. Citation searching was also conducted to identify additional studies. Two independent reviewers (G.C. and S.C.) screened titles and abstracts, assessed full texts, and resolved discrepancies through consensus or with input from a third reviewer (G.D.N.). Two researchers (G.C. and S.C.) evaluated the level of evidence of observational studies using the Melnyk and Fineout-Overholt system [25]. The search strategy is listed in Table 1.

### 2.2. Selection Criteria

Eligible studies were observational, case series, or cohort studies reporting on surgical and medical outcomes following TDH surgery. Inclusion criteria required studies to provide data on sample characteristics, surgical complications, neurological outcomes, and Freankel grades, with a minimum sample size of 20 patients and follow-up times of at least one year. The Frankel scale was chosen for this review to ensure consistency in the analysis, as it was the primary scale used across the studies included. This allowed for uniform data collection and comparison across the included studies. Studies without a clear follow-up or those with a high loss to follow-up (>20%) were excluded. Reviews, systematic reviews, and meta-analyses were assessed for references but were excluded from quantitative analysis. Criteria and definitions used in the current meta-analysis for grouping complications reported among the eligible studies are listed in Table 2.

### 2.3. Data Extraction

Two independent reviewers (G.C. and S.C.) extracted relevant data from eligible studies. The extracted variables included essential characteristics of the studies (author, publication year, country of study, follow-up time, sample size, sex distribution), sample size, mean and standard deviation of intraoperative blood loss, postoperative hospital stay, surgical time, and frequencies of neurological improvement (patient who improved at least one Frenkel grade at the last follow-up), complete hernia resection, major medical complications (stroke, pneumonia, pulmonary embolism, seizure, deep vein thrombosis, myositis ossificans, numbness, neuralgia, abdominal wall weakness, and abdominal hyperesthesia), and major surgical complications (wound infection, rebleeding, severe hemorrhage, persistent pleural effusion, hemothorax, chylothorax, incidental intraoperative dural injury, postoperative cerebrospinal fluid (CSF) leak, and CSF–pleural fistula, insufficient discectomy, residual disc, and recurrence) for both posterolateral approaches (costotransversectomy with/without pediculectomy, transpedicular, transfacet pedicle-sparing, Minimally Invasive Lateral Extracavitary Tubular Approach) and anterior approaches (thoracotomy, mini-transthoracic approach, thoracoscopy). Discrepancies in data extraction were resolved through discussion. Data were incorporated into Excel (Microsoft Corporation, Washington, DC, USA; Version 2016).

### 2.4. Statistical Analysis

A series of random-effects meta-analyses were conducted for both continuous and categorical outcomes, using the Der Simonian and Laird method to account for heterogeneity between studies. For continuous outcomes (intraoperative blood loss, postoperative hospital stay, and surgical time), mean differences (MDs) were calculated, and pooled standard deviations were computed using sample sizes and standard deviations from individual studies. The random-effects model was used to calculate summary effects and 95% confidence intervals (CIs).

For categorical outcomes (neurological improvement, complete hernia resection, major medical complications, and major surgical complications), ORs were calculated as the effect size. Log-transformed ORs were used, and their standard errors were calculated. A random-effects model was applied to account for between-study heterogeneity. Overall ORs and 95% CIs were calculated for each outcome. To assess the robustness of the meta-analysis results, we conducted sensitivity analyses for major medical and surgical complications. A one-study-removed analysis was performed, systematically recalculating the pooled ORs by removing one study at a time to evaluate the influence of individual studies on the overall effect. Additionally, we compared the results of the random-effects model with a fixed-effects model to determine the consistency of findings under different modeling assumptions. The fixed-effects model assumes homogeneity across studies, while the random-effects model accounts for potential heterogeneity. No adjustment for confounding variables was performed due to the observational nature of the included studies. Sensitivity analyses were conducted, and fixed-effects and random-effects models were applied to account for between-study variability. An OR less than 1 consistently favors the posterolateral approach, indicating either lower odds of complications or higher odds of positive outcomes such as neurological improvement or complete hernia resection.

To assess heterogeneity, Cochran’s Q statistic and the I^2^ statistic were computed for each analysis. Cochran’s Q tested for homogeneity of effect sizes across studies, while I^2^ quantified the proportion of variance due to between-study heterogeneity.

All statistical analyses were performed using inverse-variance weighting methods, and forest plots were generated to visualize the effect sizes and the variability between studies. Heterogeneity between studies was further assessed using I^2^ values, with thresholds for interpretation of low (25%), moderate (50%), and high (75%) heterogeneity. In addition, publication bias was assessed using the Egger test, which evaluates asymmetry in the funnel plot and detects potential bias across the studies included in the meta-analysis. All statistical analyses were performed using GraphPad Prism (Insight Partners, New York, NY, USA; Version 10.1.2).

## 3. Results

### 3.1. Study Selection

**Figure 1:**
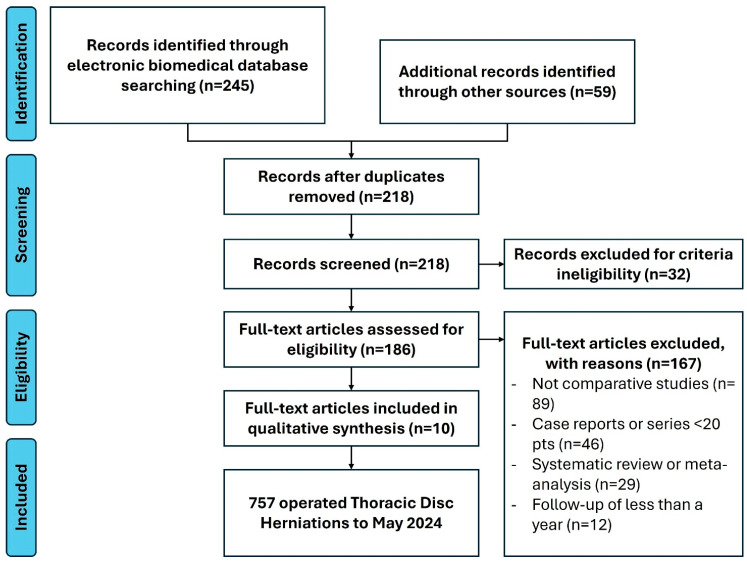
PRISMA flowchart for study selection.

Of 304 identified records, 245 were from electronic databases and 59 from manual retrieval. After excluding 32 records due to a lack of English-language full-text availability, 272 records were screened, leading to 86 abstracts. Following further exclusions, ten full-text articles, including four case series, one prospective cohort, and five retrospective cohort studies, including an overall sample of 757 patients, were included in the meta-analysis. Systematic reviews and meta-analyses were used for reference purposes and not included in statistical analyses to avoid overcrowding and duplication of values. The study selection process (PRISMA flow diagram) is detailed in Figure 1. This study was not registered on Prospero. Studies enrolled in the different statistical tests are described in Table 3. Statistical tests conducted in the study are listed in Table 4.

### 3.2. Primary Outcomes: Major Complications

**Figure 2:**
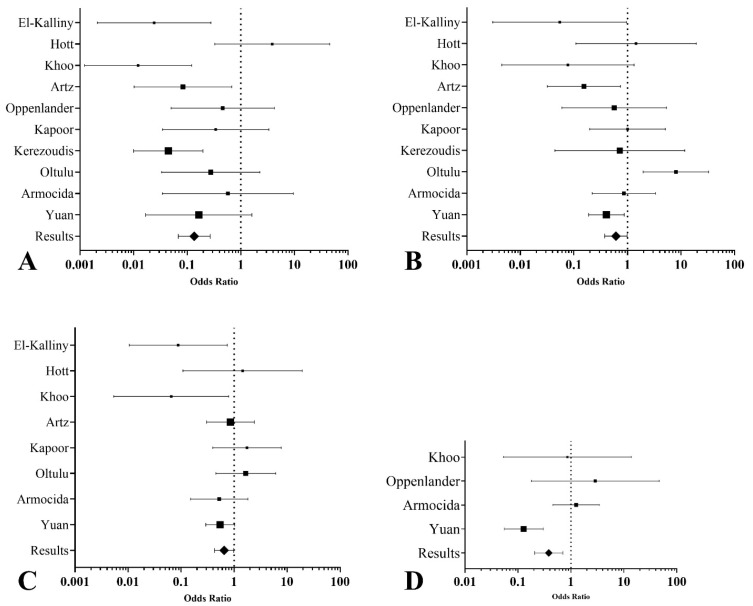
Forest plots summarizing the network meta-analysis results for thoracic disc herniation surgery outcomes. (**A**). Medical major complications: The posterolateral approach is associated with significantly lower odds of major medical complications than the anterior approach. (**B**). Surgical major complications: The posterolateral approach shows reduced odds of major surgical complications. (**C**). Neurological improvement: Odds ratios for neurological improvement suggest higher odds with posterolateral techniques. (**D**). Complete hernia resection: Posterolateral approaches demonstrate higher odds of achieving complete hernia resection than anterior approaches.

The major medical and surgical complication meta-analyses demonstrated significant differences between the posterolateral and anterior approaches. The posterolateral approach was associated with significantly lower odds of major medical complications (OR 0.14, 95% CI: 0.07 to 0.27, I^2^ = 52.18%) (Figure 2A), as well as lower odds of major surgical complications (OR 0.61, 95% CI: 0.38 to 0.99, I^2^ = 60.34%) (Figure 2B).

### 3.3. Secondary Outcomes

For secondary outcomes, the posterolateral approach showed a notable reduction in intraoperative blood loss compared to the anterior approach, with a mean difference of 398.31 mL (95% CI: −738.94 to −57.68) and substantial heterogeneity across studies (τ^2^ = 172,284.58). The posterolateral approach also significantly reduced postoperative hospital stay by an average of 2.75 days (95% CI: −3.03 to −2.47), with no observed heterogeneity (τ^2^ = 0). However, no significant difference was observed in surgical time between the two approaches, with a mean difference of −53.91 min (95% CI: −177.86 to 70.03), although high heterogeneity was present (τ^2^ = 23,549.33). The posterolateral approach was associated with significantly lower odds of incomplete hernia resections (OR 0.38, 95% CI: 0.21 to 0.71) (Figure 2C), indicating a higher likelihood of achieving complete resection than the anterior approach. Substantial heterogeneity was observed (I^2^ = 78.47%), suggesting variability between studies in how the outcomes were measured and reported. Additionally, the posterolateral approach was associated with higher odds of neurological improvement (OR 0.65, 95% CI: 0.43 to 0.99) (Figure 2D), indicating better postoperative neurological outcomes than the anterior approach.

### 3.4. Sensitivity Analysis

For major medical complications, the one-study-removed analysis showed stable results, with ORs ranging from 0.10 to 0.18 and CIs remaining consistently below 1.00, confirming the robustness of the findings. The fixed-effects model yielded an OR of 0.14 (95% CI: 0.07 to 0.27), closely aligning with the random-effects result, further validating the consistency of the outcome.

Similarly, for surgical major complications, the one-study-removed analysis produced ORs between 0.43 and 0.80, with some variability in CIs. However, the overall pattern remained stable, indicating that no single study drove the results. The fixed-effects model generated an OR of 0.61 (95% CI: 0.38 to 0.99), consistent with the random-effects model, supporting the robustness of the results despite moderate heterogeneity.

The results of the sensitivity analysis, summarized in Table S1 (Supplementary Materials), showed that no single study disproportionately influenced the overall findings, confirming the robustness of the results.

### 3.5. Heterogeneity and Publication Bias

Cochran’s Q and I^2^ statistics indicated varying degrees of heterogeneity across outcomes, with high heterogeneity for complete hernia resection (I^2^ = 78.47%). The Egger test showed no significant publication bias.3.2.

Additionally, Table S2 (Supplementary Materials) presents the pooled odds ratios, heterogeneity (I^2^), and statistical significance for each outcome variable. This table provides a justification for the observed results, highlighting the consistency of the findings across studies and addressing any observed heterogeneity through appropriate modeling.

## 4. Discussion

**Figure 3:**
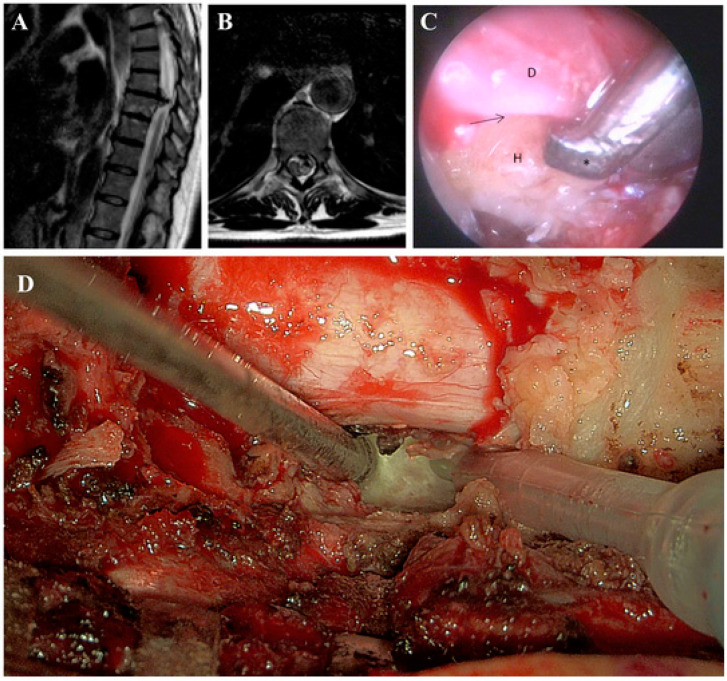
(**A**,**B**). Thoracic column MRI, sagittal and axial T2w sequence. Median calcific TDH at T8-T9 level, at the slice of the maximum anteroposterior diameter, with compression on the spinal cord and subsequent myelopathy. (**C**). Intraoperative endoscopic view. The interface (arrow) between the ventral dura (D) and the calcified herniation (H) is under control. An ultrasonic bone curette (asterisk) is used to resect the herniation while leaving the dural sac untouched. (**D**). Magnified view at the 3D-exoscope (Orbeye) of bone resection with an ultrasonic aspirator (Misonix).

TDHs, though rare, present a spectrum of symptoms from axial pain to myelopathy, often leading to delayed diagnosis and progressive neurological deficits (Figure 3A) [36]. The narrow spinal canal in this region increases the risk of spinal cord compression [12]. Surgical intervention is crucial for decompressing the spinal cord, though the choice between posterolateral and anterior approaches remains debated, with each offering distinct advantages based on the hernia’s location and severity [37].

This study compared posterolateral and anterior approaches for TDH, focusing on major complications, intraoperative blood loss, hospital stay, and neurological improvement. Despite their common use, the literature remains divided on which technique offers superior results. This meta-analysis synthesizes available data to address this gap.

The posterolateral approach was associated with significantly lower odds of major medical (OR 0.14, 95% CI: 0.07 to 0.27) and surgical complications (OR 0.61, 95% CI: 0.38 to 0.99) compared to the anterior approach. These findings suggest a safer profile with fewer complications. Sensitivity analyses confirmed the robustness of these results, with consistent ORs across models. Intraoperative blood loss (by 398.31 mL, 95% CI: −738.94 to −57.68) and postoperative hospital stay (by 2.75 days, 95% CI: −3.03 to −2.47) were significantly lower in the posterolateral group, indicating advantages in efficiency and recovery. Surgical time showed no significant difference between approaches. The posterolateral approach was associated with higher odds of neurological improvement (OR 0.65, 95% CI: 0.43 to 0.99), suggesting a potential advantage in preserving or enhancing neurological function compared to the anterior approach. Posterolateral techniques were linked to higher rates of complete hernia removal (OR 0.38, 95% CI: 0.21 to 0.71), indicating greater efficacy in achieving full resection compared to the anterior approach.

The management of TDH has advanced with the development of minimally invasive techniques. Approaches such as transforaminal endoscopic thoracic discectomy offer reduced blood loss, shorter operative times, and lower morbidity compared to traditional surgeries [38]. However, microscopic discectomy remains essential for cases involving ossification of the ligamentum flavum and severe neurological deficits requiring wider decompression [38]. While transforaminal endoscopic thoracic discectomy offers higher patient satisfaction, careful patient selection is crucial to minimize complications and optimize outcomes.

Among posterolateral approaches to thoracic spine herniations, recent research compared the transfacet, transpedicular, and costotransversectomy approaches, including the use of 45° endoscopy (Figure 3B) [8]. It was found that there were no statistically significant differences between these approaches in terms of intraoperative blood loss, postoperative complications, and herniation removal rates. Although the 45° endoscope showed potential for reducing the amount of bone resection and improving visibility, its benefits were not conclusively demonstrated in this series [8]. The results suggest that while traditional approaches remain effective, endoscopic techniques, including the use of the 45° endoscope, are still evolving and require further refinement [39]. Additional studies are needed to fully understand the long-term benefits and potential advantages of endoscopic procedures in thoracic spine surgery.

The use of advanced imaging technologies, such as the Hexoscope, has the potential to further enhance surgical outcomes in TDH procedures [8]. Similarly to the 45° endoscope, which improves visualization in confined spaces, the Hexoscope offers enhanced intraoperative imaging that may reduce the need for extensive bone resection and increase the precision of decompression (Figure 3C) [7]. As with other endoscopic innovations, further research is needed to evaluate its full impact on surgical outcomes.

Video-assisted thoracoscopic surgery (VATS) offers significant advantages over traditional open thoracotomy for TDH [40]. Studies have demonstrated that VATS reduces postoperative pain, blood loss, and hospital stays while improving shoulder girdle function and minimizing perioperative morbidity [41]. These benefits, combined with the minimally invasive nature of the procedure, make VATS a valuable alternative to open thoracotomy, which often involves greater surgical trauma and longer recovery times [41]. As highlighted by Wait et al., open thoracotomy, while providing direct visualization and access for complex cases, is associated with significantly greater postoperative pain and longer recovery times compared to VATS [40]. The higher morbidity linked to open thoracotomy underscores the advantages of minimally invasive approaches in appropriate cases [40].

Complications following TDH surgery vary depending on the approach. Posterolateral approaches consistently demonstrate lower complication rates compared to anterior techniques, which are associated with higher risks of pulmonary and neurological complications [37]. Despite the advantages of anterior approaches for central calcified herniations, the increased morbidity has led many surgeons to favor posterolateral techniques, even for complex cases [42]. These findings highlight the ongoing need for careful surgical planning and patient selection to minimize complications and optimize outcomes.

This study has several limitations. First, the data synthesis is based on retrospective studies, which introduces inherent selection bias and limits the ability to establish causality. Additionally, variations in surgical technique, surgeon experience, and patient selection criteria across the included studies may have contributed to heterogeneity in the results. The sample sizes in some studies were small, which may affect the generalizability of the findings. Moreover, the lack of long-term follow-up in many studies limits the ability to assess the durability of the surgical outcomes. It is important to acknowledge that the ASIA scale is the most globally accepted tool for spinal injury assessment. However, many of the studies included in this review utilized the Frankel scale. Future research in this field would benefit from more standardized use of the ASIA scale, ensuring broader applicability and comparability across studies. Lastly, the inclusion of various TDH types may have introduced further variability in the comparison of surgical approaches. Future large-scale, prospective trials are needed to confirm these findings and address these limitations.

This manuscript highlighted the comparative outcomes of various surgical approaches for TDH, suggesting that minimally invasive techniques may offer reduced complications and improved recovery. However, the findings point to the necessity of larger, prospective studies to validate these results and further refine surgical strategies.

## 5. Conclusions

This study highlights the advantages of posterolateral approaches for TDH, with lower complication rates, reduced blood loss, and shorter hospital stays compared to anterior techniques. While anterior approaches may still be necessary for central calcified herniations, the decreased morbidity of posterolateral techniques supports their broader use.

Future research should focus on large-scale, prospective studies to standardize surgical protocols and assess long-term outcomes. Emerging technologies, such as robotic-assisted surgery, advanced imaging like the exoscope, and minimally invasive techniques like 45° endoscopy, offer promising opportunities to enhance surgical precision and patient safety further.

## Figures and Tables

**Table 1:** PICOT search strategy of the network meta-analysis.

Frame	Mesh Terms	Search	Inclusion Criteria	Exclusion Criteria	Sources
P (patients, participants, population)	#1 “Adult spine surgery”, #2 “Thoracic disc herniation”, OR “Thoracic disc”	#1 AND #2 AND #3 AND #4 AND #5	Published in peer-reviewed journals.	Irrelevant title or abstract Irrelevant full text Editorial, reviews, meta-analysis Studies with less than 20 subjects Experimental/nonhuman studies At least one pair of comparators, Indications other than thoracic disc herniation	Databases (PubMed, Cochrane Library, ClinicalTrials.gov, Web of Science, and Scopus)
I (intervention)	#3 “Posterolateral approach”		English language		
C (comparator)	#4 “Anterior approach” OR “Thoracotomy” OR “Thoracoscopy”		Randomized controlled trials, non-randomized observational studies, cohort studies, case series		
O (outcome)	#5 “Medical Complications” OR “Surgical Complications” OR “Complete Resection” OR “Blood Loss” OR “Hospital Stay” OR Duration of Surgery”		Accurately described sample characteristics, surgical and medical complications, Frenkel grades, neurological outcomes, and Surgical parameters.		Reference list
T (time)	The search period: 1990 until December 2023				

**Table 2:** Criteria and definitions used in the current meta-analysis for grouping complications reported among the eligible studies.

Event(s)	Definition
Neurological improvement	Patients who improved at least one Frenkel grade at the last follow-up
Anterior Approach	Thoracotomy, mini-transthoracic approach, thoracoscopy
Posterolateral Approach	Costotransversectomy with/without pediculectomy, Transpedicular, transfacet pedicle-sparing, Minimally Invasive Lateral Extracavitary Tubular Approach
Surgical complications	Wound infection, rebleeding, severe hemorrhage, persistent pleural effusion, hemothorax, chylothorax, incidental intraoperative dural injury, postoperative cerebrospinal fluid (CSF) leak, and CSF–pleural fistula, insufficient discectomy, residual disc, and recurrence
Medical Complications	Stroke, pneumonia, pulmonary embolism, seizure, deep vein thrombosis, myositis ossificans, numbness, neuralgia, abdominal wall weakness, and abdominal hyperesthesia

**Table 3:** General characteristics of the eligible studies; * the value is expressed in months; † level of evidence according to Melnyk and Fineout-Overholt, 2023.

Author	Year	Country	Study Design	Recruitment Period	Patients	Anterior Approaches	Posterolateral Approaches	Neurological Improvement		Complete Hernia Resection		Medical Complications		Surgical Complications		Blood Loss		Hospital Stay		Duration of Surgery		Follow-Up *	Level of Evidence†
								ANT	POST	ANT	POST	ANT	POST	ANT	POST	ANT	POST	ANT	POST	ANT	POST		
El-Kalliny et al. [26]	1991	USA	CS	1985 to 1990	21 (7 M, 14 F), Median age 43 y.o. (SD 13.39)	8	13	6	11	-	-	3	1	1	0	-	-	-	-	-	-	3 (±12)	6
Hott et al. [27]	2005	USA	CS	1992 to 2003	20 (8 M, 12 F), Median age 61 y.o. (SD 7.75)	16	4	14	3	-	-	6	0	1	0	775	750	-	-	-	-	40.8 (±19.5)	6
Khoo et al. [28]	2011	Mexico	Rcoh	2008 to 2010	24 (9 M, 15 F), Median age 52 y.o. (SD 12.75)	11	13	8	12	11	13	7	3	3	1	295	200	5.3	5.8	175	93.75	13	4
Arts et al. [29]	2013	Netherlands	Pcoh	2005 to 2013	100 (41 M, 59 F), Median age 54 y.o. (SD 19.25)	44	33	32	25	-	-	12	1	13	2	1157	213	10.1	4.9	229	98	8.5 (±7.75)	3
Oppenlander et al. [30]	2013	USA	RCoh	1992 to 2012	56 (26 M, 30 F), Mean age 48 y.o. (SD 12.5)	39	13	-	-	39	13	6	1	5	1	1011.5	496	7	4	-	-	48 (±43.25)	4
Kapoor et al. [31]	2017	UK	Rcoh	2006 to 2014	33 (16 M, 17 F), Mean age 60 y.o. (SD 12)	22	11	11	4	-	-	5	1	6	3	1493	257	15	6.5	251	170	14.45 (±93.75)	4
Kerezoudis et al. [32]	2018	USA	CS	2012 to 2015	155 (75 M, 80 F), Mean age 53 y.o. (SD 11)	65	90	-	-	-	-	22	2	1	1	-	-	6	4	228	159	-	6
Oltulu et al. [33]	2019	Turkey	Rcoh	2007 to 2016	86 (53 M, 33 F), Mean age 56 y.o. (SD 19.25)	s68	18	58	14	-	-	12	0	4	6	390	602.78	4.87	7.17	186.79	223.12	20 (±5.4)	4
Armocida et al. [34]	2022	Italy	CS	2009 to 2019	76 (43 M, 33 F), Mean age 52 y.o. (SD 13.92)	28	48	22	42	20	32	1	1	4	6	-	-	-	-	-	-	20.25 (±28.75)	6
Yuan et al. [35]	2023	China	RCoh	2006 to 2019	186 (145 M, 41 F), Mean age 46 y.o. (SD 13.24)	63	123	2	61	39	114	3	1	17	16	947.94	716.83	15	9	180.78	163.06	75.82 (±32.5)	4

**Table 4:** Meta-analysis results for thoracic disc herniation surgery outcomes. “Pts” refers to total patients, and “Ev” to the number of events. “P1” and “P2” represent event proportions in each group. Odds ratios (ORs) and 95% confidence intervals (CIs) are provided. Statistical significance was set at *p* < 0.05. Fixed-effects and random-effects models were used. No confounding variable adjustment was performed.

Major Medical Complications											
Author	Year	Sample	Posterolateral Approach		Anterior Approach		P1	P2	Odds Ratio	95% CI	
			Pts	Ev	Pts	Ev				Lower	Upper
El-Kalliny	1991	21	13	1	8	3	0.014	0.375	0.024	0.002	0.277
Hott	2005	20	4	0	16	6	0.750	0.438	3.857	0.326	4.572
Khoo	2011	24	13	3	11	7	0.014	0.545	0.012	0.001	0.121
Artz	2013	100	33	1	44	12	0.030	0.273	0.083	0.010	0.679
Oppenlander	2013	56	13	1	39	6	0.077	0.154	0.458	0.050	4.211
Kapoor	2017	33	11	1	22	5	0.091	0.227	0.340	0.035	3.340
Kerezoudis	2018	155	90	2	65	22	0.022	0.338	0.044	0.010	0.198
Oltulu	2019	86	18	0	68	12	0.056	0.176	0.275	0.033	2.266
Armocida	2022	76	48	1	28	1	0.021	0.036	0.574	0.035	9.561
Yuan	2023	186	123	1	63	3	0.008	0.048	0.164	0.017	1.610
Results		757	366	11	364	77			**0.135**	0.068	0.268
Major Surgical complications											
			Pts	Ev	Pts	Ev				Lower	Upper
El-Kalliny	1991	21	13	0	8	1	0.008	0.125	0.054	0.003	0.961
Hott	2005	20	4	0	16	1	0.250	0.188	1.444	0.109	19.217
Khoo	2011	24	13	1	11	3	0.008	0.091	0.078	0.005	1.334
Artz	2013	100	33	2	44	13	0.061	0.295	0.154	0.032	0.739
Oppenlander	2013	56	13	1	39	5	0.077	0.128	0.567	0.060	5.353
Kapoor	2017	33	11	3	22	6	0.273	0.273	1.000	0.197	5.079
Kerezoudis	2018	155	90	1	65	1	0.011	0.015	0.719	0.044	11.713
Oltulu	2019	86	18	6	68	4	0.333	0.059	8.000	1.958	32.683
Armocida	2022	76	48	6	28	4	0.125	0.143	0.857	0.220	3.343
Yuan	2023	186	123	16	63	17	0.130	0.270	0.405	0.188	0.870
Results		757	366	36	364	55			**0.610**	0.375	0.990
Neurological improvement											
			Pts	1-Ev	Pts	1-Ev				Lower	Upper
El-Kalliny	1991	21	13	2	8	2	0.029	0.250	0.088	0.010	0.743
Hott	2005	20	4	1	16	2	0.250	0.188	1.444	0.109	19.217
Khoo	2011	24	13	1	11	3	0.014	0.182	0.065	0.005	0.794
Artz	2013	100	33	8	44	12	0.242	0.273	0.853	0.303	2.405
Kapoor	2017	33	11	7	22	11	0.636	0.500	1.750	0.396	7.733
Oltulu	2019	86	18	4	68	10	0.222	0.147	1.657	0.452	6.069
Armocida	2022	76	48	6	28	6	0.125	0.214	0.524	0.151	1.817
Yuan	2023	186	123	62	63	41	0.504	0.651	0.545	0.291	1.021
Results		546	263	91	260	87			**0.648**	0.426	0.985
Complete hernia resection											
Author	Year	Sample	Posterolateral Approach		Anterior Approach		P1	P2	Odds Ratio	95% CI	
			Pts	1-Ev	Pts	1-Ev				Lower	Upper
Khoo	2011	24	13	0	11	0	0.007	0.008	0.856	0.053	13.836
Oppenlander	2013	52	13	0	39	0	0.007	0.003	2.871	0.178	46.204
Armocida	2022	76	48	16	28	8	0.333	0.286	1.250	0.453	3.453
Yuan	2023	186	123	9	63	24	0.073	0.381	0.128	0.055	0.300
Results		338	197	25	141	32			**0.381**	0.206	0.708

## Supplementary Materials

The following supporting information can be downloaded at: https://www.mdpi.com/article/10.3390/brainsci14111062/s1, Table S1: Sensitivity analysis results for pooled odds ratios (OR) and 95% confidence intervals, with and without the inclusion of each individual study; Table S2: Meta-analysis results and justification for the pooled outcomes.
