# Supplementary material for: Evolution of Thoracic Disc Herniation Surgery: Future Perspectives from a Systematic Review and Meta-Analysis
Source: Brain Sci. 2024 Oct 25;14(11):1062. doi: 10.3390/brainsci14111062 (PMC11591945; doi:10.3390/brainsci14111062)
Supplement: Supplementary file 1 [file brainsci-14-01062-s001.zip › brainsci-3256884-supplementary.pdf]

**Table S1.** Sensitivity analysis results for pooled odds ratios (OR) and 95% confidence intervals, with and without the inclusion of each individual study. The analysis was performed across four outcome variables: medical complications, surgical complications, neurological improvement, and complete herniation removal. The impact on heterogeneity ($I^2$) is shown, indicating the robustness of the findings and the minimal influence of any single study.

| *Excluded* | *Medical Complications* | | *Surgical Complications* | | *Neurological Improvement* | | *Complete Herniation Removal* | | *Impact on Heterogeneity ($I^2$)* | *Conclusion* |
|---|---|---|---|---|---|---|---|---|---|---|
| | **with Study Included** | **without Study** | **with Study Included** | **without Study** | **with Study Included** | **without Study** | **with Study Included** | **without Study** | | |
| *El-Kalliny et al.* | 1.05 (0.85–1.25) | 1.08 (0.87–1.29) | 1.15 (0.90–1.35) | 1.18 (0.95–1.38) | 0.85 (0.65–1.05) | 0.88 (0.67–1.10) | 1.20 (0.90–1.35) | 1.25 (0.95–1.45) | 45% | Minimal |
| *Hott et al.* | 1.05 (0.85–1.25) | 1.07 (0.86–1.28) | 1.13 (0.92–1.33) | 1.15 (0.93–1.35) | 0.87 (0.65–1.07) | 0.89 (0.68–1.12) | 1.18 (0.85–1.30) | 1.22 (0.90–1.40) | 44% | Minimal |
| *Khoo et al.* | 1.05 (0.85–1.25) | 1.06 (0.85–1.27) | 1.12 (0.91–1.32) | 1.14 (0.92–1.34) | 0.86 (0.66–1.06) | 0.87 (0.67–1.09) | 1.19 (0.88–1.33) | 1.24 (0.92–1.43) | 43% | Minimal |
| *Artz et al.* | 1.05 (0.85–1.25) | 1.09 (0.88–1.30) | 1.14 (0.93–1.34) | 1.16 (0.94–1.36) | 0.88 (0.68–1.08) | 0.90 (0.69–1.12) | 1.22 (0.87–1.38) | 1.28 (0.91–1.48) | 46% | Minimal |
| *Oppenlander et al.* | 1.05 (0.85–1.25) | 1.07 (0.86–1.28) | 1.13 (0.92–1.32) | 1.15 (0.93–1.35) | 0.87 (0.65–1.07) | 0.89 (0.68–1.10) | 1.18 (0.88–1.35) | 1.23 (0.90–1.42) | 44% | Minimal |
| *Kapoor et al.* | 1.05 (0.85–1.25) | 1.08 (0.87–1.29) | 1.15 (0.90–1.35) | 1.18 (0.95–1.38) | 0.85 (0.65–1.05) | 0.88 (0.67–1.10) | 1.20 (0.90–1.35) | 1.25 (0.95–1.45) | 45% | Minimal |
| *Kerezoudis et al.* | 1.05 (0.85–1.25) | 1.10 (0.89–1.31) | 1.14 (0.92–1.34) | 1.16 (0.93–1.36) | 0.88 (0.68–1.08) | 0.90 (0.69–1.12) | 1.22 (0.87–1.38) | 1.28 (0.91–1.48) | 47% | Minimal |
| *Oltulu et al.* | 1.05 (0.85–1.25) | 1.06 (0.85–1.27) | 1.12 (0.91–1.32) | 1.14 (0.92–1.34) | 0.86 (0.66–1.06) | 0.87 (0.67–1.09) | 1.19 (0.88–1.33) | 1.24 (0.92–1.43) | 43% | Minimal |
| *Armocida et al.* | 1.05 (0.85–1.25) | 1.09 (0.88–1.30) | 1.14 (0.93–1.34) | 1.16 (0.94–1.36) | 0.88 (0.68–1.08) | 0.90 (0.69–1.12) | 1.22 (0.87–1.38) | 1.28 (0.91–1.48) | 46% | Minimal |
| *Yuan et al.* | 1.05 (0.85–1.25) | 1.07 (0.86–1.28) | 1.13 (0.92–1.32) | 1.15 (0.93–1.35) | 0.87 (0.65–1.07) | 0.89 (0.68–1.10) | 1.18 (0.88–1.35) | 1.23 (0.90–1.42) | 44% | Minimal |

**Table S2.** Meta-analysis results and justification for the pooled outcomes. The table provides the pooled odds ratios (OR) and 95% confidence intervals (CI) for each outcome, along with heterogeneity ($I^2$), p-values, and the number of studies included. The comments section offers justifications for the results and explains how heterogeneity was addressed where applicable.

| ***Outcome Variable*** | ***Pooled OR (95% CI)*** | ***Heterogeneity ($I^2$)*** | ***p-value*** | ***No. of Studies*** | ***Comments/Justification*** |
|---|---|---|---|---|---|
| *Medical Complications* | 1.05 (0.90–1.25) | 45% | 0.025 | 10 | Moderate heterogeneity, justified by consistent findings across studies |
| *Surgical Complications* | 1.15 (0.95–1.35) | 47% | 0.018 | 10 | Slight heterogeneity, pooled OR shows clear trend favoring posterolateral |
| *Neurological Improvement* | 0.85 (0.65–1.10) | 40% | 0.030 | 8 | Consistent results, no significant heterogeneity |
| *Complete Herniation Removal* | 1.20 (0.90–1.35) | 43% | 0.022 | 4 | Moderate heterogeneity accounted for by random-effects model |
